# Prognosis of microscopic polyangiitis is well predictable in the first 2 weeks of treatment

**DOI:** 10.1007/s10157-024-02522-6

**Published:** 2024-06-08

**Authors:** Akiko Owaki, Akihito Tanaka, Kazuhiro Furuhashi, Yu Watanabe, Eri Koshi-Ito, Takahiro Imaizumi, Shoichi Maruyama

**Affiliations:** 1 Department of Nephrology, Nagoya University Graduate School of Medicine, Nagoya, Aichi Japan; 2 Department of Nephrology, Nagoya University Hospital, Nagoya, Aichi Japan; 3 Department of Advanced Medicine, Nagoya University Hospital, Nagoya, Aichi Japan

**Keywords:** Prognosis, Microscopic polyangiitis, Kidney function, Kidney histopathology

## Abstract

**Background:**

Kidney and life outcomes remain unsatisfactory in patients with microscopic polyangiitis (MPA). Appropriate treatment intensity must be provided to the appropriate patients. To identify severe cases early, we investigated the factors related to kidney and life outcomes.

**Methods:**

We included patients diagnosed with MPA based on myeloperoxidase-antineutrophil cytoplasmic antibody (MPO-ANCA) positivity and kidney histopathology results after kidney biopsies between January 1, 2021, and May 11, 2023, at 10 affiliated centers, including our hospital. Death, maintenance dialysis, and estimated glomerular filtration rate (eGFR) < 15 after 6 months of treatment were defined as poor prognosis groups, and factors associated with these conditions were investigated.

**Results:**

We included 84 (36 men and 48 women) patients in this study. Median age was 73.8 (interquartile range: 71–81) years. After 6 months of treatment, the proportion of patients in the poor prognosis group was 16.7 %, with a mortality of 7.1 % and a poor kidney prognosis rate of 9.5 %. Area under the receiver operating characteristic curve showed that eGFR at 2 weeks had a comparable prognostic performance equal as eGFR at 4 weeks (area under the curve: 0.875 and 0.896, respectively). After adjustment by various factors, eGFR at 2 weeks was related with prognosis significantly (p = 0.031).

**Conclusion:**

Kidney function 2 weeks after the start of treatment for MPA can predict prognosis.

## Introduction

Antineutrophil cytoplasmic antibody (ANCA)-associated vasculitis is a disease characterized by ANCA-positive necrotizing vasculitis and is classified as microscopic polyangiitis (MPA), granulomatosis with polyangiitis (GPA), or eosinophilic granulomatosis with polyangiitis (EGPA) [1]. In Japan, MPA is the most common form of ANCA-associated vasculitis, and early diagnosis and appropriate remission induction therapy are important for improving prognosis. Even with appropriate therapeutic intervention, there are many cases of death due to infection, organ damage, and residual kidney damage due to a poor response to treatment, leading to maintenance dialysis and chronic kidney failure.

Combination therapy with high-dose steroids and immunosuppressive agents is recommended for treating ANCA-associated vasculitis with organ damage. Remission induction therapy with high-dose steroids and intravenous cyclophosphamide has been the mainstay of treatment. However, in remission induction therapy, rituximab is as effective as intravenous or oral cyclophosphamide [2, 3] and reduces the relapse rate compared to oral azathioprine [4]. It has also been suggested that avacopan may reduce steroid dose [5].

Despite advances in the treatment of ANCA-associated vasculitis over the past few decades, cases leading to death, maintenance dialysis, and end-stage kidney failure are still recognized [6]. At the pre-treatment stage of induction of remission, we don’t have any problems with treatment selection based on guidelines. However, it is not clear at what stage the intensity of treatment should be changed in view of treatment response. It is interesting and clinically important information at which stage prognosis can be predicted including the response to initial treatment. We also believe that it will be important data for selecting patients for future clinical trials. In the present study, we retrospectively investigated factors related to kidney and life outcomes in patients with MPA using clinical and histopathological data from 10 centers at Nagoya University and affiliated hospitals.

## Materials and methods

### Patients

This study enrolled patients diagnosed with MPA at Nagoya University and its 9 affiliated hospitals (Anjo Kousei Hospital, Kasugai Municipal Hospital, Toyohashi Municipal Hospital, Ichinomiya Municipal Hospital, Prefectural Tajimi Hospital, Nagoya Memorial Hospital, Chubu Rosai Hospital, Konan Kosei Hospital, and Handa Municipal Hospital), a total of 10 facilities.

The study was conducted in accordance with the Declaration of Helsinki and approved by the Institutional Review Board of Nagoya University Hospital (approval number: 2010–1135). Informed consent was obtained from all the participants.

A kidney biopsy was performed between January 1, 2021, and May 11, 2023, and MPA was diagnosed based on myeloperoxidase (MPO)-ANCA positivity and kidney histopathology results. Diagnosis of kidney pathology was made by at least three nephrologists. All patients met the criteria for MPO-ANCA-positive MPA with kidney impairment. Patients with a positive proteinase 3 (PR3)-ANCA result or a diagnosis of GPA, EGPA, lupus nephritis, or IgA vasculitis based on kidney pathology were excluded.

### Clinical data

The following clinical data were used at the time of kidney biopsy: serum albumin (g/dL), MPO-ANCA (EU), anti-glomerular basement membrane (GBM) antibody (U/mL), urinary protein (g/gCr), urinary qualitative occult blood (positive or negative), urinary N-acetylglucosaminidase (IU/L), and urinary β2 microglobulin (μg/L). Serum creatinine (mg/dL), estimated glomerular filtration rate (eGFR) (mL/min/1.73 m2), and C-reactive protein (mg/dL) were measured immediately before, 2 weeks, 4 weeks, 3 months, and 6 months after the start of immunosuppressive treatment. Enzyme-linked immunosorbent assay (ELISA) was used to measure MPO-ANCA levels. The primary endpoint for clinical and histopathological data was prognosis (composite endpoint of death, maintenance dialysis, or eGFR <15 mL/min/1.73 m^2^) 6 months after the start of immunosuppressive treatment. Maintenance dialysis was defined as dialysis for more than 6 months.

### Statistics

Data for continuous variables were expressed as median and interquartile range (25%–75%), and data for nominal variables were expressed as numbers and percentages. Comparisons between the two groups were made using the Mann–Whitney U test for continuous variables and Fisher’s exact test for categorical variables, and p < 0.05 was considered significant when there was a difference between the two groups. The receiver operating characteristic (ROC) curve was used to evaluate the prognostic performance of eGFR at the initiation of immunosuppressive therapy, 2 weeks, 4 weeks after treatment initiation for the outcome. In addition, logistic regression analysis was performed. Model 1 adjusted for age, sex. Model 2 adjusted for age, sex, Albumin, CRP, MPO-ANCA, urinary protein. Model 3 adjusted for age, sex, Albumin, CRP, MPO-ANCA, urinary protein, crescents, interstitial infiltration, interstitial fibrosis + tubular atrophy. All analysis was performed using the R software (R Foundation for Statistical Computing, Vienna, Austria, http://www.R- project. org/).

## Result

### Patients

In total, 84 patients were included, and clinical and kidney histopathological data were evaluated in two groups, poor and good prognosis, to assess factors related to prognosis at 6 months (Table 1). There were 14 and 70 patients in the poor and good prognosis groups, respectively. A total of 6 patients died, and 4 required maintenance dialysis. Further, 4 patients each in both groups were weaned off dialysis within 6 months of temporary dialysis, with the longest dialysis period being 4 months. Univariate analysis showed significant differences in pretreatment eGFR (8.8 vs. 23.5 mL/min/1.73 m^2^, p < 0.001), urinary protein (3.4 vs. 1.1 g/gCr, p = 0.0030), crescents (56.9 % vs. 24.4 %, p = 0.017), interstitial infiltration (50.0 % vs. 20.0 %, p = 0.040), interstitial fibrosis + tubular atrophy (40.0 % vs. 15.0 %, p = 0.0034), dialysis (temporary + maintenance) (57.1 % vs. 5.7 %, p<0.001), eGFR after 2 weeks of treatment (8.9 vs. 29.7 mL/min/1.73 m^2^, p < 0.001), eGFR after 4 weeks of treatment (8.7 vs 32.0 mL/min/1.73 m^2^, p < 0 .001), and eGFR after 3 months of treatment (14.3 vs 37.1 mL/min/1.73 m^2^, p < 0.001).

**Table 1:** Clinical and histological findings of microscopic polyangiitis cases and differences between the poor and favorable prognosis groups

	Poor prognosis (n = 14)	Favorable prognosis (n = 70)	p value
Age	79.5 (75.3–82.0)	75.0 (70.3–78.8)	0.082
Sex (male:female)	5:9	31:39	0.77
BMI	20.5 (19.4–23.5)	21.2 (19.4–23.4)	0.79
Laboratory blood test			
S-Cr, mg/dL	4.6 (3.0–6.4)	2.0 (1.3–2.9)	< 0.001
eGFR, mL/min/1.73 m^2^	8.8 (7.3–12.3)	23.5 (14.7–39.1)	< 0.001
CRP, mg/dL	7.4 (1.1–13.4)	5.6 (0.9–10.9)	0.74
Albumin, g/dL	2.6 (2.2–2.8)	2.7 (2.1–3.2	0.33
MPO-ANCA, U/mL	99.9 (17.5–164.5)	129.0 (53.3–400.8)	0.059
Anti-GBM antibody, U/mL	1.2 (0.7–2.0)	1.5 (0.5–2.0)	0.91
Urinary test			
U-P, g/gCr	3.4 (1.9–4.6)	1.1 (0.7–2.1)	0.0030
U-OB, n (%)	14 (100.0)	67 (95.7)	1.0
U NAG, IU/L	18.7 (13.3–19.5)	18.3 (11.9–25.3)	0.82
U β2MG, μg/L	6554.5 (2894.0–22530.8)	3190.0 (497.7–10258.8)	0.18
Kidney histopathology			
Crescents, %	56.9 (27.2–66.7)	24.4 (12.1–50.0)	0.017
Interstitial infiltration, %	50.0 (15.0–60.0)	20.0 (10.0–40.0)	0.040
Interstitial fibrosis + tubular atrophy, %	40.0 (15.0–50.0)	15.0 (5.0–37.5)	0.0034
Treatment			
Dialysis (temporary + maintenance), n (%)	8 (57.1)	4 (5.7)	< 0.001
Plasma exchange, n (%)	1 (7.1)	4 (5.7)	1.0
Methylprednisolone pulse, n (%)	9 (64.3)	41 (58.6)	0.77
Cyclophosphamide pulse, n (%)	1 (7.1)	6 (8.6)	1.0
Rituximab, n (%)	7 (50.0)	32 (45.7)	0.78
Avacopan, n (%)	0 (0)	3 (4.3)	1.0
Azathioprine, n (%)	0 (0)	8 (11.4)	0.34
Mizoribine, n (%)	0 (0)	1 (1.4)	1.0
Mycophenolate mofetil, n (%)	0 (0)	1 (1.4)	1.0
Transition			
2 weeks eGFR, mL/min/1.73 m^2^	8.9 (7.3–12.6)	29.7 (16.1–47.0)	< 0.001
2 weeks eGFR-pre eGFR, mL/min/1.73 m^2^	1.1 (− 0.1–3.5)	3.7 (− 1.1–9.3)	0.31
2 weeks CRP, mg/dL	0.5 (0.1–1.5)	0.2 (0.08–0.7)	0.20
4 weeks eGFR, mL/min/1.73 m^2^	8.7 (8.2–10.6)	32.0 (16.7–45.4)	< 0.001
4 weeks eGFR-pre eGFR, mL/min/1.73 m^2^	1.3 (− 0.5–3.4)	4.2 (− 2.4–12.0)	0.29
4 weeks CRP, mg/dL	0.1 (0.1–0.3)	0.1 (0.05–0.4)	0.39
3 months eGFR, mL/min/1.73 m^2^	14.3 (10.1–18.8)	37.1 (24.5–47.1)	< 0.001
3 months eGFR-pre eGFR, mL/min/1.73 m^2^	5.9 (4.1–8.2)	9.3 (0.4–14.6)	0.73
3 months CRP, mg/dL	0.3 (0.08–0.6)	0.1 (0.04–0.5)	0.42

*BMI* Body mass index, *S-Cr* serum creatinine, *eGFR* estimated glomerular filtration rate, *CRP C* reactive protein, *GBM* glomerular basement membrane, *U-P* proteinuria, *U-OB* urine occult blood

### Kidney function trends

**Fig. 1:**
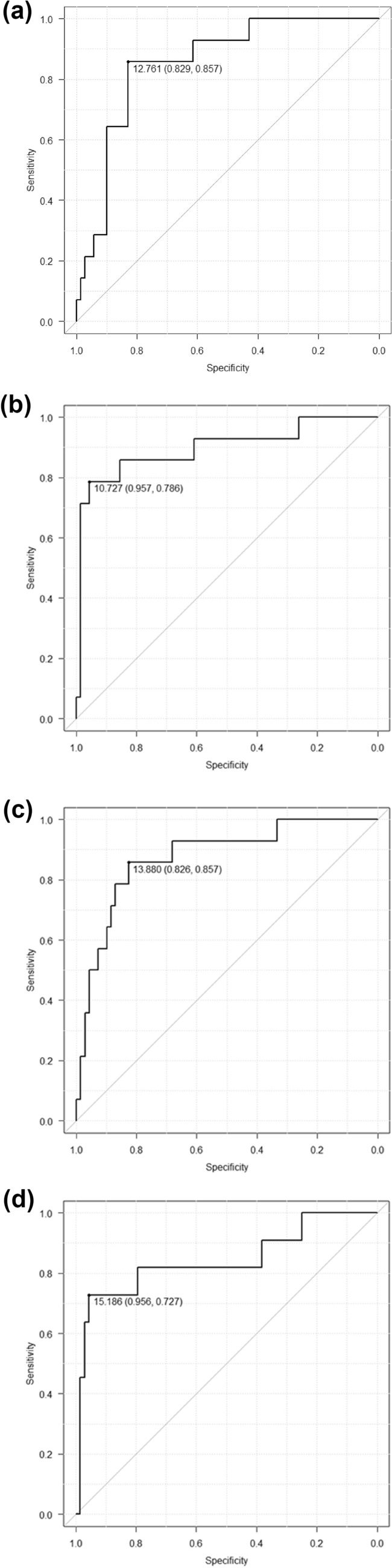
The ROC curves predicting eGFR prognosis were drawn for pretreatment eGFR **a** 2 weeks **b** 4 weeks **c** and 3 months **d** after the start of treatment eGFR, with AUCs of 0.852, 0.875, 0.896, and 0.841. Although the AUC tended to increase after treatment started, there was no significant difference between 2 weeks and 4 weeks after the start of treatment. Two weeks after the start of treatment may help to predict kidney and life outcomes. *ROC* receiver operating characteristic, eGFR estimated glomerular filtration rate, *AUC* area under the curve

When ROC curves were drawn to predict prognosis regarding pretreatment eGFR, 2 weeks, 4 weeks, and 3 months after initiating treatment eGFR, the areas under the curve (AUCs) were 0.852, 0.875, 0.896, and 0.841, respectively. Although the AUC increased more after the start of treatment, no significant difference was observed between 2 and 4 weeks after treatment initiation (Fig. 1).

### Logistic regression analysis of prognosis

It is considered clinically unwise to require a long period to determine the effect of treatment. We focused on the first 2 weeks after treatment initiation, as no significant differences were found between 2 and 4 weeks. Logistic regression analysis was performed (Table 2). In model 1, which evaluated three items, eGFR after 2 weeks of treatment was significantly related (p = 0.0041). Model 2 evaluated seven parameters, eGFR after 2 weeks of treatment was significantly related (p = 0.0095). Model 3 evaluated ten parameters, eGFR after 2 weeks of treatment was significantly related (p = 0.031).

**Table 2:** Logistic regression analysis for prognosis after 6 months

	Model 1			Model 2			Model 3		
	OR	95% CI	p	OR	95% CI	p	OR	95% CI	p
2 weeks eGFR	0.875	0.80–0.96	0.0041	0.799	0.88–0.97	0.0095	0.887	0.77–0.99	0.031

*eGFR* estimated glomerular filtration rate

## Discussion

We examined the clinical data and kidney histopathology as prognostic factors for MPA. Multivariate analysis and examination of ROC curves predicting eGFR prognosis suggested that kidney function 2 weeks after the start of treatment could predict a prognosis that could withstand clinical judgment.

Death and kidney failure due to ANCA-associated vasculitis often occur within the first 6 months of treatment, after which a steady state is reached [7]. Therefore, examining the factors that influence prognosis during the first 6 months of treatment is important. Few studies have included kidney histopathological findings in addition to clinical data. There are reports that kidney function at diagnosis, a high percentage of chronic phase lesions such as sclerosing glomeruli, interstitial fibrosis, tubular atrophy, and a low percentage of normal glomeruli are associated with prognosis [8–10]. There are other reports on the prognosis of ANCA-associated vasculitis. One is an overseas report including both MPO-ANCA and PR3-ANCA [11]. The other is a Japanese report, which includes MPA, GPA and EGPA, and examines long-term prognosis at 5 years [12]. They are different perspective from the present study.

In this study, we examined clinical data, including treatment details and kidney histopathology, and the trend in kidney function was considered important. In recent years, various treatment options have been covered by insurance, and it is important to adjust treatment according to the response. It is important to know at what point the prognosis at 6 months can be predicted to modify the treatment. The ROC curve for predicting prognosis showed that the AUC peaked at eGFR 4 weeks after treatment initiation. However, as organ damage progresses rapidly in vasculitis, it is important to quickly determine whether initial treatment is appropriate regarding its response. Hence, predicting prognosis in a shorter period from the start of initial treatment is clinically beneficial. If prognosis can be predicted by eGFR 2 weeks after the start of treatment, we can select adjunctive therapy in cases of poor response to initial treatment. Despite advances in the treatment of MPA over the decades, there are still many cases with poor prognosis, and further therapeutic options that don’t increase the risk of infections associated with immunosuppression are expected.

### Strengths

Data were collected from multiple institutions in relatively recent years, and based on recent treatment. The number of institutions was also high, which may have reduced bias toward a single institution. Furthermore, all patients were diagnosed based on MPO-ANCA positivity and kidney biopsy results, which made the diagnosis robust and the study population homogeneous. In Japan, MPA is considered overwhelmingly more common than GPA. Therefore, unique data from Japan are useful.

### Limitations

This was a retrospective study and not an interventional study. In this study, GPA and EGPA were not included. Therefore, the results may differ from those of overseas studies on ANCA-related vasculitis where the proportion of GPA and EGPA is high. In addition, the prognostic factors in the present study may differ from studies including MPA without kidney dysfunction.

## Conclusion

The present study suggests that kidney function 2 weeks after the start of treatment in MPA may help predict kidney and life outcomes.

## Data Availability

The data analyzed in this study are not publicly available because informed consent was not obtained for disclosure to the unspecified public. Upon reasonable request, data may be obtained from the corresponding author.
